# Evaluation of the diagnostic performance of colposcopy in the diagnosis of histologic cervical intraepithelial neoplasia 2+ (CIN2+)

**DOI:** 10.1186/s12885-022-10030-7

**Published:** 2022-08-29

**Authors:** Namkha Dorji, Sangay Tshering, Sonam Choden, Meera Chhetri, Damanti Bhujel, Tshering Wangden, Birendra Pradhan, Pema Choden Bhutia, Ugyen Tshomo

**Affiliations:** 1 Department of Obstetrics and Gynaecology, Jigme Dorji Wangchuck National Referral Hospital, Thimphu, Bhutan; 2 Department of Pathology and Laboratory Medicine, Jigme Dorji Wangchuck National Referral Hospital, Thimphu, Bhutan; 3 Colposcopy Clinic, Gyeltsuen Jetsun Pema Mother and Child Hospital, Jigme Dorji Wangchuck National Referral Hospital, Thimphu, Bhutan

**Keywords:** Colposcopy, Papanicolaou test, Uterine cervical pathology, Cervical cancer screening tests, Histopathology, Women’s health

## Abstract

**Background:**

Colposcopy is a tool for triaging screen positive women regardless of method used for cervical cancer screening. The objective of this study was to evaluate the diagnostic performance of colposcopy in the diagnosis of histologic cervical intraepithelial neoplasia 2+ (CIN 2+) at Jigme Dorji Wangchuck National Referral Hospital (JDWNRH), Thimphu, Bhutan.

**Methods:**

This cross-sectional study was conducted from March 2021 to August 2021 among 299 women who availed colposcopy services at the colposcopy clinic of JDWNRH, Bhutan. Women included in this study were either screen positive (Pap smear) or were suspected to have invasive cancer; they underwent colposcopy and a cervical biopsy irrespective of colposcopy impression. This histopathologic assessment was considered as the gold standard test for the diagnosis of cervical intraepithelial neoplasia (CIN) or invasive cancer.

**Results:**

The mean age of the study participants was 43 years (ranges, 25–76 years). The sensitivity, specificity and accuracy of senior colposcopists to diagnose histologic CIN 2+ were 80.0% (95% CI 59.30, 93.17), 71.07% (95% CI 62.13, 78.95), and 72.60% (95% CI 64.61, 79.65), and for junior colposcopists were 59.46% (95% CI 42.10, 75.25), 76.72% (95% CI 67.97, 84.04), and 72.55% (95% CI 64.76, 79.45) respectively. The overall sensitivity, specificity, and accuracy of colposcopy to diagnose histologic CIN 2+ were 66.67% (95% CI 53.66, 78.05), 73.73% (95% CI 67.63, 79.23), and 72.24% (95% CI 66.79, 77.24) respectively.

**Conclusions:**

In this study, the senior and junior colposcopists had a comparable colposcopic accuracy to diagnose histologic CIN 2+, whereas senior had a higher sensitivity but a lower specificity than junior colposcopists.

## Background

In 2020, cervical cancer was the fourth most frequently diagnosed cancer and fourth leading cause of death amongst women with an estimated 604,000 new cases and 342,000 deaths worldwide [1]. It is the most common cancer, and the leading cause of cancer deaths among Bhutanese women [1]. Persistent infection of the cervix with high risk-human papillomavirus (HPV) has been established as a necessary (but not sufficient) cause for developing cervical cancer [2]. Cervical cancer is preceded by a long period of recognisable cytological and histological changes providing an opportunity for early detection by screening procedures [3]. The highly effective primary (HPV vaccine) and secondary preventive measures makes the cervical cancer a nearly completely preventable disease, justifying the World Health Organization’s (WHO) ambitious target of eliminating cervical cancer by 2030 through 90–70-90 strategies [1, 4]. The prevention of cervical cancer plays an important role to achieve the sustainable development goal (SDG), both for health (SDG 3) and gender equality (SDG 5) [5].

The screening methods available for cervical cancer include cytology - conventional Pap smear and liquid-based cytology (LBC), and dual staining to identify p16 and Ki-67; visual methods like visual inspection with acetic acid or with Lugol’s iodine (VIA/VILI); and molecular nucleic acid amplification tests (NAAT) such as high-risk HPV DNA NAAT and mRNA [6]. Bhutan adopted conventional cytology as the screening method for women in the age group of 25–65 years at 3 year intervals, since the year 2000 [7]. The ASCCP recommends management of cervical screening abnormalities based on the assessment of immediate CIN3+ risk. The management can be either expediated treatment, expediated colposcopy, or return in 1-, 3-, and 5- years [8].

Colposcopy is an important adjunctive procedure in the screening of cervical cancer; it helps in studying the size and location of precancer lesions (high grade CIN). We then either confirm presence of precancer by taking biopsy or advice treatment without confirmation (see and treat). However, the performance of colposcopy in the diagnosis of cervical pathology has wide inter-observer variation depending on duration of the experience of colposcopists [9]. Many studies have reported the accuracy of colposcopy in diagnosing precancerous and other lesions of cervix in various clinical settings, in different groups of patients with various presentation and using different outcome measures [9–11]. The objective of this study was to evaluate the performance of colposcopy in the diagnosis of histologic CIN2+ at Jigme Dorji Wangchuck National Referral Hospital (JDWNRH), the national referral hospital of Bhutan.

## Method

### Study population

This cross-sectional study was conducted from March 2021 through August 2021 among Bhutanese women who underwent colposcopy at JDWNRH. They were recruited by convenience sampling method. Married or sexually active women between the ages of 25 and 76 years with abnormal cytology result or women with gynecological complaints such as postcoital bleeding, intermenstrual bleeding, postmenopausal bleeding and unhealthy cervix who consented to undergo colposcopy and biopsy (either with punch biopsy or loop electro-surgical excisional procedure (LEEP) were included. Women with obvious cervical cancer, with history of LEEP, cold knife conization (CKC), and cryotherapy, treated for invasive cervical cancer with surgery, and/or chemoradiation therapy, and known pregnancy or puerperium were excluded.

The data on socio-demographic details of women were collected using an interviewer administered questionnaire after obtaining written informed consent. The data variables were age, place of current residence, educational status, marital status, occupation, age at first sexual intercourse, age at first childbirth, parity, and monthly income in Ngultrum.

Bhutan is located in the eastern Himalayas with a total population of 7,74,830 with female population consisting of 46.3% as of 2015 [12]. The health services are provided through a three-tiered structure: national and regional referral hospital at the tertiary level, general or district hospitals at secondary level, and basic health units (BHU), sub-posts and outreach clinics (ORCs) at the primary level.

### Conventional pap smear

Conventional Pap smear services are provided at all three levels of health care services in Bhutan. The specimens are taken by trained female health assistants or medical officers in the field, and transported to the laboratory in the standard method. Examination of cytology slides are done by the trained cyto-technicians. The suspicious slides are cross checked and confirmed by cyto-pathologists before issuing reports. Conventional Pap smear is done in the standard methods, and reported as per the 2014 Bethesda System for Reporting Cervical cytology including terminologies as- negative for intraepithelial lesion or malignancy (NILM), atypical squamous cells which is further subdivided into atypical squamous cells of undetermined significance (ASC- US) and atypical squamous cells, cannot exclude high-grade squamous intraepithelial lesion (ASC-H), low-grade squamous intraepithelial lesion (LSIL), high-grade squamous intraepithelial lesion (HSIL) and/or squamous cervical carcinoma (SCC).

### Colposcopy and histopathology

**Fig. 1:**
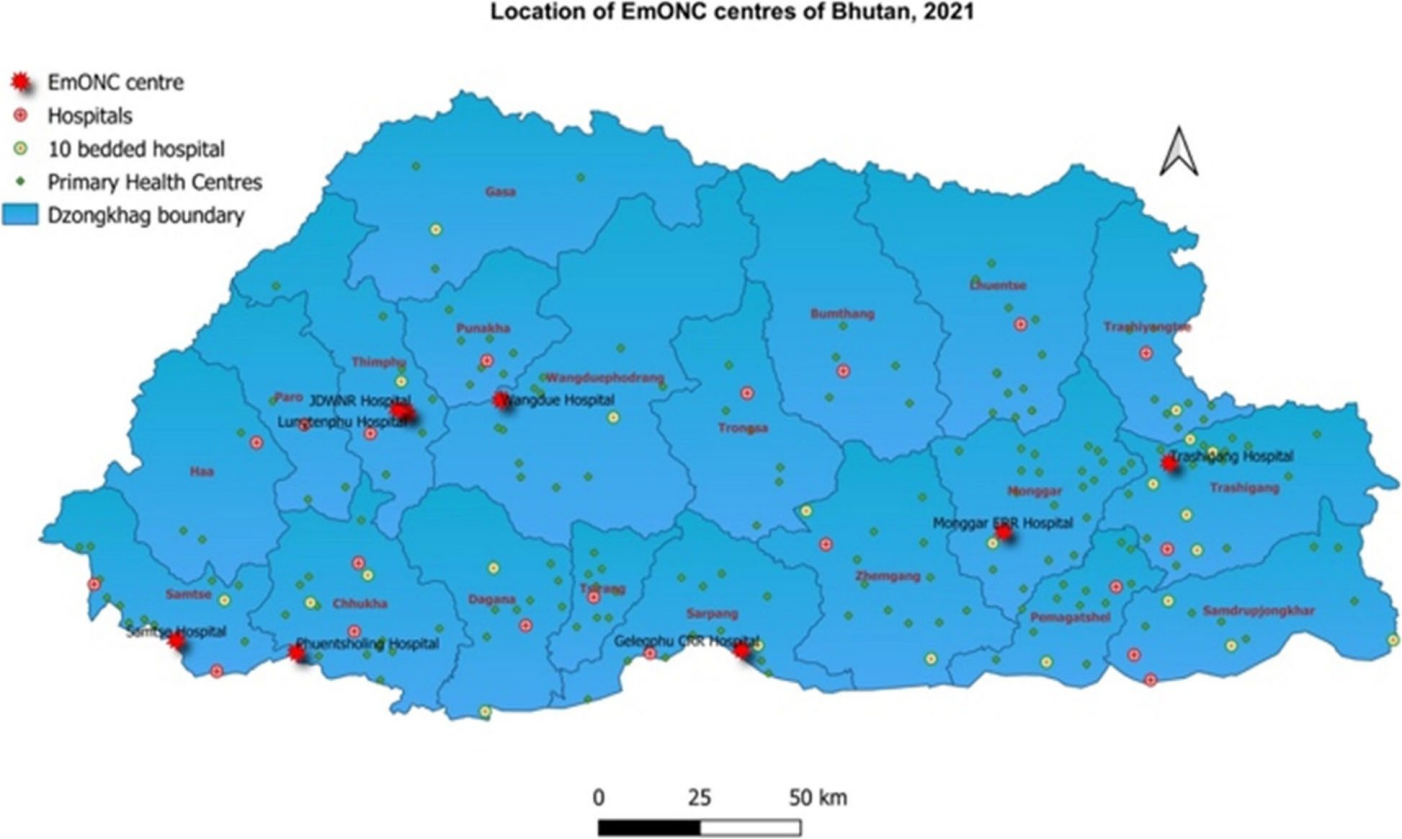
Map of Bhutan showing the sites of comprehensive EmONC centers with pink asterisk (Used with permission from the Policy & Planning Division, Ministry of Health, Bhutan)

The routine colposcopy services are provided by the Obstetrician-Gynaecologist at the comprehensive emergency obstetrics and neonatal care (EmONC) centres (Fig. 1), and during colposcopy camp services conducted in the secondary and primary health centres. Those cases with abnormal cytology reports are referred for colposcopy as per the national cervical cancer screening guideline, which is based on 2011 International Federation of Cervical Pathology and Colposcopy (IFCPC) [13]. As colposcopy clinics are conducted on fixed days (either once or twice a week), patient are routed based on prior appointment dates co-ordinated between the health workers in the field and colposcopy nurse.

In this study, the colposcopy was performed by five Obstetrician-Gynaecologists, who had undergone some form of training abroad (Thailand). Two colposcopists with working experience as colposcopists for more than 10 years were categorized as “senior colposcopists”, and other three colposcopists with less than 10 years of working experience as colposcopists are categorized as “junior colposcopists”. The video colposcope (***EDAN C3A***) using 6–12 magnification was used. The standard steps described in the International Agency for Research on Cancer manual of colposcopy were followed [14], and the colposcopy findings were documented according to the International Federation for Cervical Pathology and Colposcopy nomenclature, and Swede score calculated [15, 16]. The colposcopy finding was described as normal, low-grade lesion (LGL), high grade lesion (HGL), and suspicious of invasion. Punch biopsy from the densest acetowhite areas near squamo-columnar junction (SCJ), and multiple punch biopsies in cases of normal colposcopy were taken using Kevorkian or Baby Tischler minibite biopsy forceps. Excisional treatment with LEEP was performed under total intravenous anaesthesia (TIVA) in the operation theatre complex of the hospital.

The cervical tissue specimens obtained from the patients were placed in 10% phosphate-buffered formalin and transported to the histology unit, Department of Pathology and Laboratory medicine, JDWNRH. The slides were constructed from 3 mm sections of formalin-fixed paraffin-embedded (FFPE) blocks and stained with Mayer’s Hematoxylin (HIMEDIA) and Eosin yellow (HIMEDIA) in the Sakura Tissue-Tek DRS 200 slide Stainer as per the standard operating protocol. The slides were then viewed under binocular light microscopy by two Pathologists, blinded to the colposcopy and the Pap results. The consensus report was provided by using the terminology of the WHO Classification of Female Genital Tumors, 5th edition [17]*.*

### Statistics and data analysis

Data were double entered, validated using EpiData (Version 3.1 for entry and version 2.2.2.183 for analysis, EpiData Association, Odense, Denmark), and analysed using STATA (version 13.1, StataCorp LP USA). The numerical variables were presented as mean and standard deviation, and the categorical variables were presented as frequency and percentage using descriptive commands. Sensitivity, specificity, and accuracy were used to assess the diagnostic performance of colposcopy to diagnose histologic CIN 2+. Chi-square test was used to analyze the relation between colposcopy and histologic results.

CIN 2+ (disease positive) included histopathologic diagnoses of CIN II, CIN III, CIS and invasive carcinoma, whereas CIN 2- (disease negative) included histopathologic diagnoses of normal, inflammation, and CIN I. For calculating performance of colposcopy, HGL and suspicious of invasion in the colposcopy were taken as colposcopy positive, whereas normal and LGL were taken as colposcopy negative.

## Results

### Socio-demographic characteristics of study population

In this cross-sectional study conducted for a period of 6 months from March 2021 to August 2021 at the colposcopy clinic of JDWNRH, 299 women fulfilling inclusion criteria were recruited. The mean age of the study participants was 43.23 ± 11.01 year (range, 25–76 years). Their mean age at coitarche was 20.56 ± 4.21 years (range, 12–37 years), and the mean age at first child birth was 20.79 20.79 ± 6.71 years (range, 13–39 years). One hundred and eighty-eight (62.88%) study participants were living in Thimphu, and 264 (88.29%) were married. One hundred and fifty women (50.17%) had no education, 157 (52.51%) were housewives, and 84 (28.09%) had a monthly income of less than Nu.10,000. These details of socio-demographic characteristics are shown in table 1.

**Table 1:** Socio-demographic characteristics of women who underwent colposcopy at JDWNRH^a^ (*n* = 299)

Variables	Frequency (n)	Percentage (%)
**Age in years** (mean ± SD)/Range	(43.23 ± 11.01)/25–76	
25–30	50	16.72
31–40	77	25.75
41–50	91	30.77
51–76	80	26.76
**Residence**		
Thimphu	188	62.88
Outside Thimphu	111	37.12
**Marital status**		
Married	264	88.29
Divorced/widow/unmarried	35	11.71
**Educational status**		
No education	150	50.17
Educated	149	49.83
**Occupation**		
House wife	157	52.51
Civil servant	40	13.38
Private office	21	7.02
Businesswoman	35	11.71
Farmer	35	11.71
Others (nuns, dependent)	11	3.68
**Monthly income (in Ngultrum**^**b**^)		
< =10,000	84	28.09
11,000-20,000	74	24.75
21,000-30,000	45	15.05
31,000-40,000	19	6.35
41,000-50,000	22	7.36
> =51,000	35	11.71
Dependents	20	6.69
**Parity**^**c**^		
Nulliparous	18	6.02
Multiparous	281	93.98
Age at coitarche (mean ± SD)/Range	(20.56 ± 4.21)/12–37	
Age at first child birth (mean ± SD)/Range	(20.79 ± 6.71)/13–39	

^a^Jigme Dorji Wangchuck National Referral Hospital; ^b^Bhutanese currency; SD, standard deviation; ^c^number of pregnancies that have crossed 24 weeks of gestation

### Clinical characteristics of study population

The most common cytology result was ASCUS of 86 (28.76%), followed by inflammatory changes 64 (21.41%), LSIL 45 (15.05%), normal 31 (10.37%), ASC-H 23 (7.69%), AGC 18 (6.02%), and HSIL 12 (4.01%). Ninety-eight (32.78%) had normal colposcopy, followed by LGL 97 (32.44%), HGL 83 (27.76%) and suspicion of invasion 21 (7.02%). In the histopathologic diagnosis, 98 (32.78%) had chronic cervicitis, followed by normal 99 (33.11%), CIN II/III/CIS 47 (15.71%), CIN I 39 (13.05%), and invasive cancer 16 (5.35%). These details are shown in Table 2.

**Table 2:** Distribution of study participants according to cytology, colposcopy and histology results (*n* = 299)

	Frequency (n)	Percentage (%)
**Cytology results**		
Normal	31	10.37
Inflammatory	64	21.41
ASCUS	86	28.76
AGC	18	06.02
LSIL	45	15.05
ASC-H	23	07.69
HSIL	12	4.01
Suspicious of SCC	20	6.69
**Colposcopy results**		
Normal	98	32.78
LGL	97	32.44
HGL	83	27.76
Suspicious of microinvasive cancer	21	7.02
**Histopathology results**		
Normal	99	33.11
Chronic cervicitis	98	32.78
CIN I	39	13.05
CIN II/III/CIS	47	15.71
SCC	16	5.35
**Colposcopist**		
Senior	146	48.8
Junior	153	51.2

*CIN* Cervical intraepithelial neoplasia, *NILM* Negative for intraepithelial lesion or malignancy, *ASCUS* Atypical squamous cells of undetermined significance, *AGC* Atypical glandular cells, *LSIL* Low-grade squamous intraepithelial lesion, *ASC-H* Atypical squamous cells, cannot exclude high-grade squamous intraepithelial lesion, *HSIL* High-grade squamous intraepithelial lesion, *CIS* Carcinoma in situ, *SCC* Squamous cervical carcinoma, *LGL* Low grade lesion, *HGL* High grade lesion

### Cytology-histopathology correlation

Discrepancy of cytology-histology correlation (CHC) is shown in Table 3. One hundred and thirty-one (43.81%) had agreement, 89 (29.77%) had minor variance, 44 (14.72%) had minor (under call/overcall) discrepancy and 35 (11.70%) had a major (under call/overcall) discrepancy.

**Table 3:**
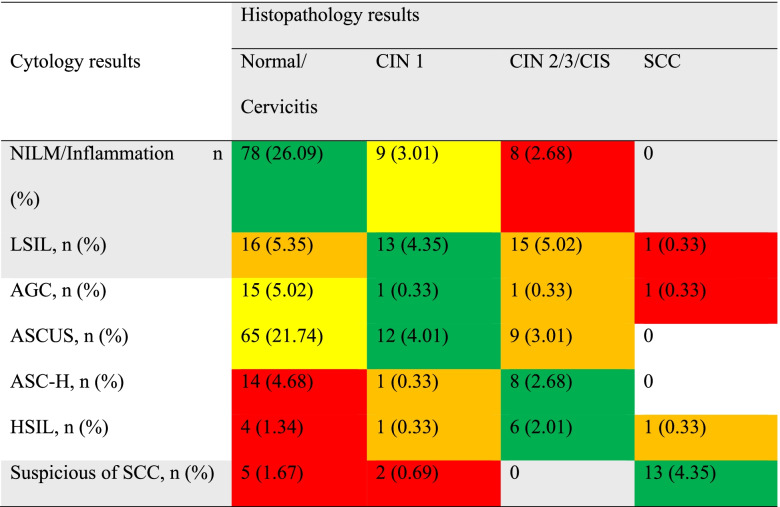
Discrepancy assessment grid (over- and under call refers to the cytology interpretation (*n =* 299).

Green—agreement; yellow—minor variance; orange—minor (under call/over call) discrepancy; and red—major discrepancy (under call/over call) cases. Percentage calculated for all included cases. *CIN* Cervical intraepithelial neoplasia, *NILM* Negative for intraepithelial lesion or malignancy, *ASCUS* Atypical squamous cells of undetermined significance, *AGC* Atypical glandular cells, *LSIL* Low-grade squamous intraepithelial lesion, *ASC-H* Atypical squamous cells, cannot exclude high-grade squamous intraepithelial lesion, *HSIL* High-grade squamous intraepithelial lesion, *CIS* Carcinoma in situ, *SCC* Squamous cervical carcinoma.

### Diagnostic performance of colposcopy to diagnose histologic CIN 2 +

The diagnostic performance of colposcopy to diagnose histologic CIN 2+ cervical pathology is shown in Table 4. The accuracy (72.60% versus 72.55%) between senior and junior colposcopists was almost equal, whereas the specificity (71.07% versus 76.72%) of junior was higher than senior colposcopists. The sensitivity (80.00% versus 59.46%) was higher in senior as compared with junior colposcopists.

**Table 4:** Performance of colposcopy in the diagnosis of histopathologic CIN 2 + ^a^

	Sensitivity % (95% CI)	Specificity % (95% CI)	Accuracy % (95% CI)
Senior colposcopists	80.00 (59.30, 93.17)	71.07 (62.13, 78.95)	72.60 (64.61, 79.65)
Junior colposcopists	59.46 (42.10, 75.25)	76.72 (67.97, 84.04)	72.55 (64.76, 79.45)
Overall	66.67 (53.66, 78.05)	73.73 (67.63, 79.23)	72.24 (66.79, 77.24)

^**a**^CIN2+ includes CIN II, CIN III, CIS & invasive cancer; CI, confidence interval

## Discussion

In this cross-sectional study among 299 women, we found out that the colposcopy had an accuracy of 72.24% to diagnose histologic CIN2+, sensitivity of 66.67% and specificity of 73.73% with a statistically significant correlation (*p* < 0.001) between colposcopy positive and histologic CIN 2+ which is comparable with studies done elsewhere [10].

The cytologic-histologic agreement in our study was 43.81%, minor discrepancies of 29.77%, and major discrepancies of 11.73% which is a poor performance as compared with studies in Russia and India [18, 19]. A study on cervical cytology-histology correlation based on the American society of cytopathology guideline (2017) in Russia noted 57.9% cytology-histology agreement, 7.6% major discrepancies, and 34.15% minor discrepancies. Thirteen (7.6%) discrepancy was due to colposcopy sampling error, and 16.8% (*n =* 42) was due to Pap smear sampling error [18]. Another retrospective review of 92 cases with minor or major cytology-histology discrepancy, 39.1% (*n =* 36) were due to error in interpretation, 31.5% (*n =* 29) were due to sampling error, and 19.5% (*n =* 18) were due to cervical smear error [19].

The possible reasons for such a high cytology-histology discordance probably could be due to Pap smear sampling error, colposcopy sampling error, and error in the interpretation of cytology. In Bhutan, there is no quality assurance, supervision, or refreshers course for female health assistants who are taking samples for Pap smear. In the absence of adequate cyto-pathologists in the country, cyto-technicians are involved in interpretation of cytology results. Other reasons for high cytology-histology discordance could be due to punch biopsies missing the site of lesion. In this study, LEEP was performed in 16.7% of the study participants, whereas 83.3% underwent punch biopsies.

Many studies have reported the accuracy of colposcopy in diagnosing precancerous and other lesions of cervix in various clinical settings, in different groups of patients with various presentation and using different outcome measures [9–11]. The performance of colposcopy in the diagnosis of cervical pathology has wide inter-observer variation depending on duration of the experience of colposcopists. Women’s age, postmenopausal status, and type 3 transformation zone were found to be associated with diagnostic inaccuracies of colposcopy-directed punch biopsy [20]. The colposcopic sensitivity (93.5% versus 92.2%, *p* < 0.001) and accuracy (85.9% versus 80.0%, *p <* 0.001) of senior colposcopists to diagnose CIN 2+ lesions were significantly higher than those of junior colposcopists. The specificity (76.0% versus 72.4%, *p* = 0.290) of senior colposcopists was slightly higher (but no statistical significance) than junior colposcopists [9]. In contrast, our study found that the sensitivity (80.0% versus 59.6%) senor colposcopists with more than 10 years’ experience was higher than junior colposcopists with less than 10 years’ experience. However, senior had lower specificity (71.07% versus 76.72%) than senior, with almost comparable accuracy (72.60% versus 72.55%). Our explanation for lower sensitivity among senior colposcopists to diagnose histologic CIN 2+ lesions could be due to lack of refreshers course and some amount of knowledge or skills attrition over the years. Among two senior colposcopists, one was trained in 2000 and the other one in 2009. Among three junior colposcopists, on was trained in 2013, and two were trained in 2018. Two colposcopists, one from each category attended online refreshers course in 2020 organized by IFCPC.

In this study, the overall sensitivity, specificity, and accuracy of colposcopy positive to diagnose histologic CIN 2+ were 66.67% (95% CI 53.66, 78.05), 73.73% (95% CI 67.3, 79.23), and 72.24% (95% CI 66.79, 77.24) respectively. These appear lower than similar studies conducted elsewhere in the world [9–11, 21]. A similar study in Bangladesh had found the sensitivity of colposcopy to detect CIN 2+ was 50% (95% CI 28, 72) and specificity was 94% (95% CI 88, 98) [11]. In a similar study done in Turkey, the sensitivity and accuracy of colposcopy were 92 and 96% respectively, and the specificity was 67% [10]. A study in China, the sensitivity, specificity and accuracy of colposcopy to diagnose HSIL+ were 70.2, 75.1 and 72.9% respectively [9]. In a systematic review and meta-analysis of thirty-two papers comprising 7873 paired punch/definitive histology, the pooled sensitivity of colposcopy directed punch biopsy to diagnose CIN 2+ was 91.3% (95% CI 85.3–94.9%) and the specificity was 24.6% (95% CI 16.0–35.9%). The authors have concluded that the high sensitivity and low specificity might be the result of verification bias, and the high sensitivity is probably a spurious finding as most of the studies restricted to excision mainly to women with a positive punch biopsy [22].

In Bhutan, colposcopists follow the standard steps of colposcopy procedure, documentation and interpretation [14, 16]. However, in practice, situation arises where colposcopists bypass or don’t adhere to the recommended steps such as not applying normal saline, not waiting for 1 minute after application of normal saline and/or acetic acid, and acetic acids are not freshly prepared, Lugol’s iodine is out of stock. Although 2 out of 5 colposcopists involved in this study has more than 10 years of colposcopy experience, many of us did not avail any refreshers course. Some of these practical reasons might have contributed towards low performance of colposcopy in this study.

This is the first study on colposcopy conducted in our country which gives baseline data, and way forward for improvement. The study design being a cross-sectional study, cervical biopsies taken from colposcopic normal cervices, and pathologist blinded to Pap smear and colposcopy results are the major strengths. With the insight gained from this study, we would like to make some recommendations to improve the accuracy of colposcopy in our setting: adhering strictly to the standardised colposcopy steps, regular refreshers course to the colposcopists to upgrade their knowledge and improve skills, and the consumables such has freshly prepared acetic acid and Lugol’s iodine has to be made available at all times.

## Conclusion

From this study, we conclude that the sensitivity, specificity and accuracy of colposcopy in our clinic was lower than similar studies in other countries. The sensitivity among senior colposcopists was higher than junior, specificity was lower, and the accuracy was comparable between senior and junior colposcopists.

We need to take corrective measures towards improving the performance of colposcopy such as performing colposcopy by strictly adhering to the standardised colposcopy steps, regular refreshers courses for the colposcopists, regular supervision and regular quality assurance so that Bhutanese women get the best services they deserve.

## Data Availability

The datasets generated and/or analyzed during the current study are not publicly available due personal information protection, patient privacy regulation, and medical institutional data regulatory policies, etc., but are available from the corresponding author on reasonable request and with permission of the Research Ethics Board of Health, Ministry of Health, Thimphu, Bhutan.

## Abbreviation

ASCCP: American Society of Colposcopy and Cervical Pathology
ASC-H: Atypical squamous cells, cannot exclude high-grade squamous intraepithelial lesion
ASC-US: Atypical squamous cells which is further subdivided into atypical squamous cells of undetermined significance
BHU: Basic Health Unit
CI: Confidence Interval
CIN 2 +: Cervical Intraepithelial Lesion 2+
CKC: Cold Knife Conization
DNA: Deoxy ribonucleic Acid
EmONC: Emergency obstetrics and neonatal care
FFPE: Formalin-fixed paraffin-embedded
HPV: Human Papilloma Virus
HSIL: High-grade squamous intraepithelial lesion
IFCPC: International Federation of Cervical Pathology and Colposcopy
JDWNRH: Jigme Dorji Wangchuck National Referral Hospital
LEEP: Loop Electrosurgical Excision Procedure
LBC: Liquid Based Cytology
LSIL: Low-grade squamous intraepithelial lesion
NAAT: Nucleic Acid Amplification Test
NILM: Negative for intraepithelial lesion or malignancy
ORC: Outreach Clinic
RNA: Ribonucleic acid
SCC: Squamous cervical carcinoma
SDG: Sustainable Development Goal
TIVA: Total intravenous anaesthesia
VIA: Visual Inspection with Acetic acid
VILI: Visual Inspection with Lugol’s Iodine
WHO: World Health Organization
